# Effects of adding thoracic spine exercises to routine soccer training on spinal alignment and mobility in professional male soccer players: a randomized controlled study

**DOI:** 10.1186/s13102-026-01633-9

**Published:** 2026-03-12

**Authors:** Kazım Bayram, Derya Özer Kaya

**Affiliations:** 1 Institute of Health Sciences, Department of Physiotherapy and Rehabilitation, İzmir Kâtip Çelebi University, İzmir, Türkiye; 2 Faculty of Health Sciences, Department of Physiotherapy and Rehabilitation, İzmir Kâtip Çelebi University, İzmir, Türkiye

**Keywords:** Exercise, Thoracic spine, Sports, Soccer

## Abstract

**Background:**

Thoracic spine mobility is essential for trunk stability, rotational movement, and athletic performance in soccer players. This study aimed to investigate whether the addition of a six-week thoracic spine exercise program to routine soccer training affects sagittal thoracic alignment, segmental spinal mobility, and thoracic rotation in professional male football players, compared with routine training alone.

**Methods:**

In this parallel-group randomized controlled trial, 42 professional male soccer players (mean age 20.85 ± 3.41 years) were randomly assigned in a 1:1 ratio to either the exercise or control group (*n* = 21/group). The exercise group performed thoracic spine exercises three times per week for six weeks in addition to their routine training, while the control group continued routine training without receiving any intervention. Pre- and post-intervention assessments included spinal alignment and mobility in the sagittal and frontal planes (using the Valedo^®^Shape system), the Matthias test, and thoracic rotation angles (measured with a smartphone Compass application). Two-way repeated-measures ANOVA and post-hoc tests were used to analyze group (G) × time (T) interactions and within-group changes. Effect sizes (Cohen’s d) were calculated.

**Results:**

The primary outcome, sagittal thoracic kyphosis angle measured using the Valedo^®^Shape system, did not demonstrate a significant G × T interaction or T effect, indicating no differential change between groups over the intervention period. As a key secondary outcome, bilateral thoracic rotation angles showed significant G × T interactions, with the exercise group demonstrating substantially greater improvements than the control group. The thoracic spine exercise program demonstrated high feasibility, with 100% participation and no intervention-related adverse events reported. Effect sizes were large for thoracic rotation angles (d = 1.00-1.46) and Matthias test inclination angle (d = 1.42–1.97).

**Conclusion:**

The six-week thoracic spine exercise program improved spinal alignment, segmental mobility, and thoracic rotation in professional male soccer players. These findings suggest that coaches, sports science specialists, and physiotherapists could consider incorporating thoracic spine exercises into training and rehabilitation programs to support spinal function and optimize athletic performance.

**Trial registration:**

ClinicalTrials.gov (NCT07253415), retrospectively registered on 19 November 2025. https://clinicaltrials.gov/study/NCT07253415.

**Supplementary Information:**

The online version contains supplementary material available at 10.1186/s13102-026-01633-9.

## Introduction

Soccer is a dynamic and multifaceted sport that involves complex movement patterns, including high-speed running, sudden stops and changes of direction, jumping, collisions, and trunk rotation [1–3]. The spine serves not only as a central component of the skeletal system but also as a dynamic structure essential for trunk stability, postural control, balance, and efficient force transmission [4, 5]. In athletes, maintaining coordination between body segments, ensuring efficient functioning of the kinetic chain, and sustaining high-level motor control all require optimal spinal function [6]. In high-contact and high-intensity sports such as soccer, the spine is exposed to substantial repetitive loads due to its anatomical structure, which can result in microstructural stress accumulation, postural deviations, and segmental dysfunctions over time, ultimately reducing performance and increasing the risk of injury [2].

The thoracic spine, often referred to as the “Cinderella” region of the spine, functions not only as a passive postural support but also as a key regulator of intersegmental movement, rotational mobility, and trunk stability [7, 8]. Its unique structural characteristics, including its regional width, articulations with the ribs, and functional position within the spinal column, distinguish it from the cervical and lumbar regions [9]. However, because the thoracic spine forms the rib cage together with the sternum, ribs, costovertebral joints, and associated ligamentous structures, its anatomical configuration limits thoracic range of motion (ROM) [10, 11]. Furthermore, the thoracic spine plays a critical role in maintaining vertical posture, ensuring head stability, increasing kinesthetic feedback, and enabling controlled and high-quality movements in the distal segments [9, 12]. A review of the current literature indicates that interventions for trunk control and spinal health in soccer players predominantly focus on the core and lumbar regions, while exercises targeting the thoracic region are not systematically addressed [8, 13–15].

Football is a multidimensional sport characterized by the repetitive performance of complex, high-intensity motor skills such as sprinting, rapid changes of direction, jumping, shooting, and passing [16]. Although performance in these actions is often evaluated primarily in terms of lower extremity strength, trunk kinematics and spinal function are fundamental determinants of force transmission efficiency and movement economy [17, 18]. During sprinting and rapid directional changes, rotational control of the trunk and thoracic spine mobility enable effective redirection of ground reaction forces and efficient transfer of momentum into a new plane of movement [19–22]. When thoracic mobility or trunk control is insufficient, compensatory increases in movement may occur at the lumbar spine and pelvis, accompanied by asymmetrical load distribution in the lower extremities. Such compensatory strategies may not only impair performance but also increase the risk of injury [21, 22]. Moreover, in football-specific technical skills such as shooting and passing, axial rotation of the trunk plays a key role in transferring momentum from the trunk to the lower limb, thereby directly influencing kicking performance [23–25]. In addition, the ability to maintain adequate trunk stability in frequently encountered unbalanced game situations is critical for both performance preservation and injury prevention [26].

Recently, research on spinal exercise interventions and their effects on spinal structure, segmental ROM, and functional mobility has expanded and gained clinical relevance [14, 27, 28]. Exercise programs specifically targeting the thoracic region have been shown to support spinal segmental integrity and improve mobility in both the sagittal and frontal planes [8, 12, 29]. Limited trunk rotation flexibility has been significantly associated with shoulder and elbow injuries in athletes who rely heavily on the upper extremities (e.g., shooters, tennis players), and restricted thoracic rotation may represent a risk factor for such injuries [30]. Proper trunk rotation sequencing during complex movements such as throwing reduces mechanical load on the shoulder joint and promotes intersegmental balance, thereby lowering injury risk [31]. Furthermore, limitation of thoracic spine mobility may contribute to the development of shoulder impingement syndrome and reduce force production in movements such as shoulder abduction [32, 33]. Thoracic spine exercises have been shown to increase thoracic mobility in the sagittal and transverse planes [34], expand the thoracic ROM in individuals with thoracic kyphosis, and reduce both thoracic slope and spinal inclination angles [35]. Thoracic stabilization exercises have also been reported to reduce thoracic and lumbar tilt angles in the sagittal plane while increasing mobility [12, 36, 37]. It has been reported that exercises targeting the thoracic spine produce beneficial effects across various athletic populations. For example, in tennis players, such programs increase thoracic mobility without altering the thoracic inclination angle in the sagittal plane, whereas in swimmers, they lead to a direct and measurable increase in thoracic mobility [38, 39]. In recent years, thoracic region-specific exercise approaches have been shown to improve postural control as well as upper and lower extremity performance [40]. However, studies that directly investigate the effects of thoracic spine–specific exercise interventions on spinal alignment and segmental mobility remain scarce. This paucity of evidence constitutes a notable gap in the literature, particularly in athletic populations exposed to high mechanical loading [12, 39].

Previous studies have suggested that neuromuscular control of the spine and intersegmental stability can be modified within approximately four weeks of targeted spinal stabilization exercises [41, 42]. Extending the intervention beyond this initial adaptation period has been shown to consolidate and maintain functional improvements [43]. Accordingly, a six-week intervention period was selected to ensure sufficient time for neuromuscular and postural adaptations to develop and stabilize, while remaining feasible within a professional athlete population.

Considering the current literature, it is evident that further research is needed to investigate and compare the effects of thoracic spine exercises on spinal alignment and mobility. Therefore, this study aimed to investigate whether the addition of a six-week thoracic spine exercise program to routine soccer training affects sagittal thoracic alignment, segmental spinal mobility, and thoracic rotation in professional male football players, compared with routine training alone. The primary outcome of this study was the sagittal thoracic kyphosis angle (°) measured using the Valedo^®^Shape system. Secondary outcomes included bilateral thoracic rotation and other spinal alignment and mobility parameters. In this study, spinal alignment was defined as segmental angular parameters assessed in the neutral posture using the Valedo^®^Shape system in the sagittal and frontal planes. Spinal mobility was defined as the dynamic angular ROM exhibited by the thoracic spine during active movements, including sagittal-plane flexion–extension, frontal-plane lateral flexion, and transverse-plane thoracic rotation. We hypothesized that the exercise group would demonstrate a greater improvement in sagittal thoracic kyphosis angle compared with the control group after a 6-week thoracic spine exercise program. Additionally, it was hypothesized that thoracic spine exercises would result in more pronounced and consistent improvements in thoracic spine rotation and other spinal alignment and mobility parameters, leading to significant differences between the exercise and control groups.

## Methods

### Study design and setting

This study was reported in accordance with the Consolidated Standards of Reporting Trials (CONSORT) 2025 guidelines. A two-group, randomized controlled trial design with a 1:1 allocation ratio was used to investigate the effects of thoracic spine exercises on spinal alignment and mobility. The trial was conducted in Izmir, Turkey between April and August 2024 at the training facilities of a professional soccer club.

### Participants

A total of 49 professional male soccer players from a Second League soccer club based in Izmir, Turkey were assessed for eligibility. Inclusion criteria were being a professional male soccer player over the age of 18, voluntary participation in the study, and regular attendance at team training sessions. Exclusion criteria included a history of major sports injuries requiring surgery or injuries that caused time loss, musculoskeletal deformities of the lower and upper extremities, a history of injury within the last 3 months, and spinal trauma within the last 6 months. All participants were prospectively recruited and enrolled prior to baseline assessment and randomization. No retrospective enrollment or post hoc inclusion occurred.

### Ethical considerations

The study protocol was approved by the Izmir Katip Celebi University Institutional Review Board (Approval Number: 0187, April 2024). All players were provided with detailed information regarding the nature and scope of the study, potential risks, and anticipated benefits prior to participation. Written informed consent to participate in the study was obtained from all participants prior to enrollment. The study was conducted in accordance with the principles of the Declaration of Helsinki.

### Patient and public involvement

Participants were not involved in the design of the study, selection of outcome measures, conduct of the trial, or reporting of the results. No changes were made to the trial design, eligibility criteria, interventions, outcomes, or statistical analyses after the trial commenced.

### Outcome measures

The primary outcome of this study was sagittal thoracic kyphosis angle (°) assessed using the Valedo^®^Shape system (Hocoma, Idiag, Fehraltorf, Switzerland). The secondary outcomes included other spinal alignment and mobility parameters assessed using the Valedo^®^Shape, bilateral thoracic rotation angles measured using smartphone-based Compass app, and the segmental sagittal-plane angles and global trunk inclination angle obtained during the Matthias test.

### Sample size

Prior to the study, a power analysis was conducted using data obtained from a previously published study [12] that reported the mean and standard deviation values of the sagittal thoracic kyphosis angles (T1-T12, degrees) measured with the Valedo^®^Shape system in two comparison groups. Based on these data the effect size was calculated as 0.871. The required sample size was then calculated for a split-plot analysis of variance (ANOVA) design using G*power software version 3.1.9.2, with a two-sided significance level of α = 0.05 and s statistical power of 0.85. A power level of 0.85 was selected to reduce the risk of Type II error, considering the relatively small and homogeneous population of professional athletes, in whom recruitment feasibility is limited and clinically meaningful changes in thoracic alignment might otherwise remain undetected. The analysis indicated that a minimum sample size of 40 participants (*n* = 20 per group) would be required.

### Randomization and blinding

Participants were randomly assigned to groups using a computer-generated random allocation sequence with simple randomization. Allocation was implemented through a computer-based system that ensured concealment of the allocation sequence until assignment. The individual responsible for participant enrollment and assignment did not have access to the randomization sequence. Outcome assessments were performed by an assessor who was not involved in the exercise intervention and was blinded to group allocation. Statistical analyses were conducted by a biostatistician who was blinded to the study aims and group assignments.

### Study procedures

All players participated in a detailed information session outlining the interventions and assessment procedures. Baseline assessments were conducted prior to randomization, and follow-up outcome assessments were completed immediately after the 6-week intervention period. Both groups continued to participate in the routine soccer training program planned by the club’s technical staff, which was implemented 5–6 days per week and lasted approximately 90–120 min per session. Routine soccer training included standardized warm-up activities, technical practices (e.g., passing, shooting, ball control, one-on-one play), game structure and tactical organization (e.g., positional plays, transition plays, set plays), conditioning components (aerobic and anaerobic endurance, agility, speed, strength), and cool-down protocols. In addition to routine training, the exercise group performed the thoracic spine exercise program, whereas the control group received no additional intervention. All assessments and interventions were conducted under standardized conditions at the club’s indoor gym and soccer field at the same time of day. All exercise sessions were supervised by a certified physiotherapist. All participants in the exercise group began and completed the intervention on the same dates. The intervention consisted of a total of 18 exercise sessions. The study was carried out during the competition period, and players in both groups were monitored to ensure comparable physical activity levels. Adverse events and harms related to the thoracic spine exercise program were monitored throughout the intervention period by the supervising physiotherapist. Participants were instructed to report any pain, discomfort, or injury occurring during training sessions.

### Intervention: thoracic spine exercise training program

Following baseline evaluations, players in the exercise group performed a structured thoracic spine exercise training program in addition to their routine soccer training, three times per week for six weeks. Each exercise session lasted approximately 30–40 min, resulting in a total of 18 sessions. The exercise group continued to participate in the routine training program. planned by the club technical team, implemented 5 to 6 days a week and lasting approximately 90–120 min per session.

The thoracic spine exercise program consisted of six exercises targeting thoracic mobility, strength, motor control, and stability: [1] thoracic spine extension exercise with foam roller [2], thoracic extension flexibility exercise [3], thoracic spine rotation exercise in a plank position [4], thoracic rotation exercise in a quadrupedal position [5], side-plank thoracic rotation (open book) exercise, and [6] bird-dog exercise. All exercises were performed with controlled movement speed, within pain-free ranges of motion, and required active participation of the participants. Movement quality and postural alignment were continuously monitored throughout the sessions, and verbal feedback was provided when necessary to ensure correct exercise execution. All exercise sessions were conducted face-to-face in a group-based format under the direct supervision of a certified physiotherapist with 12 years of experience in sports physiotherapy.

In this study, the thoracic exercise training program was designed according to the principle of progressive overload, with systematic increases in repetition and set numbers and a gradual escalation of resistance levels over time, with the aim to increase thoracic ROM and support segmental movement capacity associated with spinal alignment in the sagittal and frontal planes. The exercise program followed a predefined standardized structure for all participants; however, exercise progression was individually tailored based on adaptation levels.

Exercise intensity was monitored using the Borg Rating of Perceived Exertion (RPE) scale, and all exercises were planned and performed at a moderate intensity level (RPE 11–13). Decisions regarding progression were based on participants’ perceived exertion (RPE), maintenance of correct movement form, and post-exercise recovery responses. If pain, excessive fatigue, or impaired movement control was observed, progression of the relevant exercise was postponed or the current level was maintained. In particular, for the open book and bird-dog exercises, elastic resistance bands were introduced from the second week onward, progressing from light to moderate resistance in accordance with weekly improvements in motor control [44–46].

**Fig. 1:**
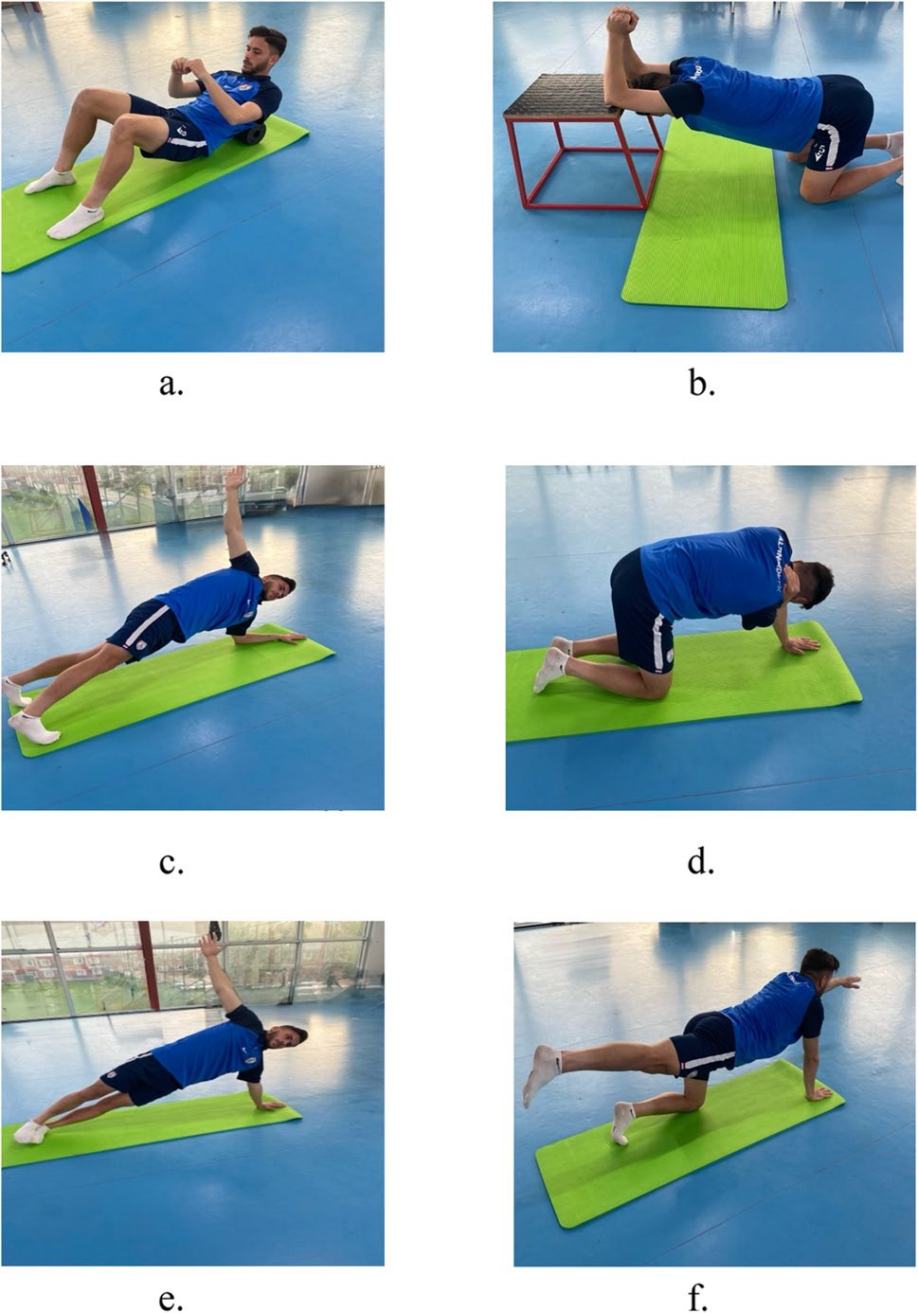
Thoracic spine exercises. **a** Thoracic spine extension exercise with a foam roller, **b** Thoracic extension flexibility exercise, **c** Thoracic spine rotation exercise, **d** Thoracic rotation exercise in quadrupedal position, **e** Open book exercise, **f** Bird-dog exercise

Adherence to the exercise program was prospectively monitored using attendance logs for each session. When a participant was unable to attend a scheduled session, a make-up session was arranged within the same week to maintain program continuity. All participants in the exercise group completed the planned 18 exercise sessions, resulting in 100% compliance. No missed sessions occurred during the intervention period. The specific thoracic spine exercises are illustrated in Fig. 1 [35, 41, 47, 48]. Sufficient details of the intervention protocol are provided in the main text and Supplementary Material 2 to allow replication. In addition, a complete exercise manual with detailed instructions is provided in the Supplementary Material 1.

### Assessments

#### Spinal alignment and mobility evaluation

Spinal alignment and mobility of the players were assessed using the Valedo^®^Shape. This device evaluates posture and mobility of the thoracic (T1–T2 to T11–T12) and lumbar (T12–L1 to the sacrum) spine in the sagittal and frontal planes. The Valedo^®^Shape is a non-invasive, computer-assisted measurement system that provides valid and reliable results comparable to radiographic analysis and does not involve radiation exposure [49, 50]. The average ICCs were reported to be higher than 0.80 for the global sagittal angle of the lumbar and sacral spine and trunk and the total sagittal mobility of the thoracic and lumbar spine and trunk [50, 51]. Postural alignment and spinal mobility were evaluated in the sagittal and frontal planes. Before the assessment, players were given detailed explanations and demonstrations of each evaluation position and were allowed to practice the movements once to ensure familiarity. After the correct evaluation position was assumed, the spinous processes of the spine were marked as anatomical reference points. The Valedo^®^Shape device was then moved downward by the assessor along all spinous processes, beginning from the C7 vertebra to approximately the S3 level. During the measurements, participants were instructed to maintain the required position until the assessment was completed [50–52].

For the purposes of this study, the primary outcome, sagittal thoracic kyphosis angle, was defined as an angular parameter reflecting the sagittal-plane inclination of the thoracic spine during a static measurement performed while the participants were standing in an upright, neutral, and relaxed posture, and it was recorded in degrees (°).

During the measurements, angular increases in the direction of flexion were considered positive values, whereas changes in the direction of extension were considered negative values.

For the sagittal plane evaluation, measurements were obtained in three positions: neutral posture, maximum flexion, and maximum extension. For the frontal plane evaluation, measurements were taken in the neutral posture as well as during right and left lateral flexion.

In the final part of the assessment, the Matthias test was performed to evaluate spinal alignment changes in response to sustained upper limb loading. Participants stood upright with their shoulders flexed to 90°, holding weights in both hands for 30 s. Measurements were obtained immediately after the weights were lifted and again after the 30-second holding period, without releasing the weights. The load was individualized according to body weight: 1.5 kg for players under 55 kg, 2 kg for those between 56 and 70 kg, 2.5 kg for those between 71 and 85 kg, and 3 kg for those above 86 kg. During the Matthias test, segmental sagittal-plane angles of the thoracic, lumbar, and sacral spine, as well as the global trunk inclination angle, were recorded in degrees (°). Data were transmitted via Bluetooth to a computer screen in real time, automatically calculated, and recorded using dedicated software [53].

#### Evaluation of thoracic rotation

An iPhone (Apple Inc., Cupertino, CA, USA) and the Compass application were used to measure thoracic rotation. Players were asked to sit, cross their hands in front of their chest, and hold a stick between their arms at shoulder height. The smartphone was placed at the T1-T2 level, and the measurement started from the position from the 0° position. While maintaining contact with the phone, players performed active thoracic rotation, and the resulting angular value was recorded. Players were then asked to return to 0°, and the test was concluded. This method has been reported to demonstrate high reliability (ICC: 0.87). Measurements were repeated three times, and the average value was included in the analysis [54].

Three weeks prior to the start of the study, thoracic angle measurements were conducted using the Valedo^®^Shape device and the iPhone Compass application, three times per week over three consecutive weeks. The purpose was to establish the assessor’s intra-rater reliability before the main data collection phase.

### Statistical analysis

All statistical analyses were conducted using SPSS Statistics, Version 26 (IBM Corp., Armonk, NY). Descriptive data are presented as mean ± standard deviation (SD). The normality of data distribution was assessed using the Shapiro-Wilk test. Pre-test differences between the exercise and control groups for all variables were evaluated using one-way ANOVA, and homogeneity of variances was confirmed using Levene’s test. Two-way repeated-measures ANOVA was used to analyze main effects and interactions for all variables. Analyses were conducted on participants who completed the study and had complete pre- and post-intervention data (complete-case analysis). Two participants (one from each group) withdrew after randomization and did not provide post-intervention data; therefore, an intention-to-treat analysis with imputation was not performed. Given the low and balanced attrition between groups and the absence of post-intervention data for these participants, a complete-case approach was considered appropriate. No subgroup analysis was performed. No interim analyses were planned, and no stopping guidelines were applied during the study.

To limit the risk of multiplicity, a single primary outcome (sagittal thoracic kyphosis angle) was prespecified a priori. All other outcomes were considered secondary and were analyzed in an exploratory framework. For variables showing significant main or interaction effects, Bonferroni-adjusted post hoc comparisons were applied where appropriate. Effect sizes were reported to facilitate interpretation of the magnitude and practical relevance of the observed effects.

Effect sizes were interpreted as small (ηp² < 0.01), medium (ηp² = 0.02–0.1), and large (ηp² > 0.1) [55, 56]. When significant main or interaction effects were observed, post-hoc analyses were performed using paired t-tests with Bonferroni correction to determine differences between pre-test and post-test values within each group. Within-group effect sizes (Cohen’s d) were calculated as d = (M_1_ − M_2_)/σ_pooled_, with values of 0.00–0.19 considered negligible, 0.20–0.49 small, 0.50–0.79 medium, and ≥ 0.80 large [55, 57]. The significance level (α) was set at 0.05.

A sensitivity analysis was conducted to evaluate the robustness of the findings to missing data. For the two participants who withdrew after randomization and had no post-intervention measurements, post-test values were conservatively imputed using a baseline observation carried forward approach. The primary outcome (sagittal thoracic kyphosis) and key secondary outcomes (bilateral thoracic rotation angles) were reanalyzed using the same two-way repeated-measures ANOVA framework.

## Results

**Fig. 2:**
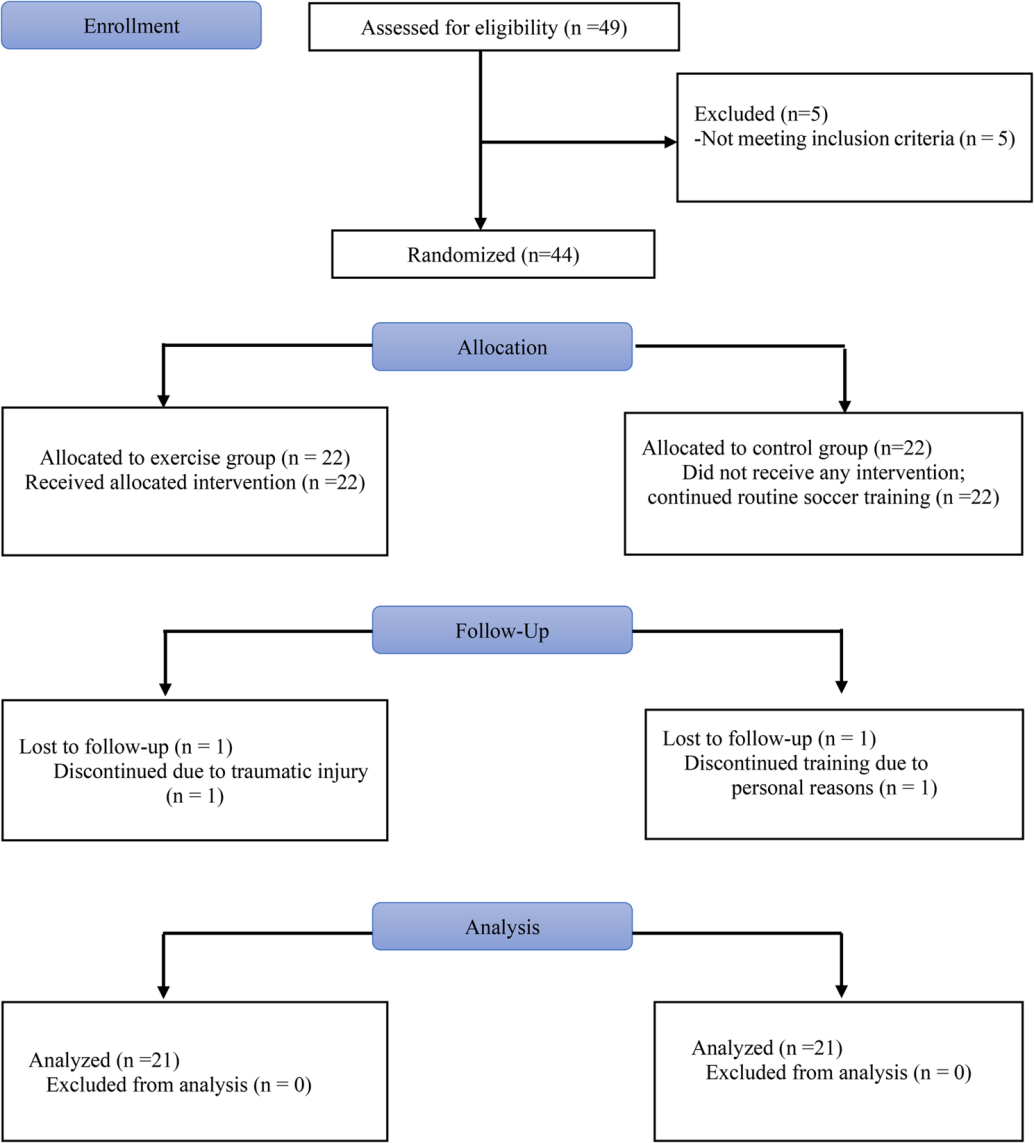
The study flow diagram

Participants were recruited and enrolled between April 2024 and August 2024. A total of 49 professional male soccer players were assessed for eligibility. Five players were excluded from the study for not meeting the inclusion criteria. Following baseline assessments, 44 players were randomly assigned to the exercise group (*n* = 22) or control group (*n* = 22). During the study period, one participant from the exercise group withdrew due to a traumatic injury sustained during routine team training, and one participant from the control group withdrew for personal reasons. As a result, a total of 42 participants (*n* = 21 per group) completed the study and were included in the final analyses. The study flow diagram is presented in Fig. 2.

The mean age of the participants was 20.85 ± 3.41 years. Outcome data were complete for all participants included in the analyses at baseline and post-intervention assessments. All primary and secondary outcomes were analyzed according to group allocation. The training-related injury was not associated with the intervention. No other adverse events or unintended effects were reported in either group during the study period. The intervention was delivered as intended.

Baseline demographic and physical characteristics of the participants are presented in Table 1. There were no significant differences between the exercise and control groups at baseline in terms of age, height, body weight, body mass index, or soccer playing experience (*p* > 0.05). The spinal alignment and mobility sub-parameters of the participants are presented in Table 2.

**Table 1:** Baseline demographic and physical characteristics of the participants

	Exercise Group (*n* = 21)	Control Group (*n* = 21)	*p*-value
Characteristic			
Age (years)	20.76 ± 3.40	20.95 ± 3.51	0.85
Height (cm)	179.42 ± 6.49	180.23 ± 6.48	0.68
Body weight (kg)	71.71 ± 5.35	72.09 ± 7.18	0.84
Body Mass Index (kg/m^2^)	22.26 ± 1.10	22.16 ± 1.52	0.79
Soccer playing experience (years)	9.95 ± 3.12	10.00 ± 2.56	0.95

Independent samples t-test. Values are presented as mean ± standard deviation. cm: centimeters, kg: kilograms, kg/m^2^: kilograms per square meter

**Table 2:** Spinal alignment, segmental mobility, and thoracic rotation angles before and after six weeks of thoracic spine exercises in exercise and control groups

	Exercise Group (*n* = 21)		Control Group (*n* = 21)		
	Pre-test Mean ± SD	Post-test Mean ± SD	Pre-test Mean ± SD	Post-test Mean ± SD	Two-way ANOVA (T and G x T): *p*, F, ηp^2^
Sagittal Plane (Angle)					
Thoracic	39.71 ± 6.50	40.33 ± 8.81 d = 0.078	42.48 ± 8.13	41.67 ± 7.72 d = 0.10	T: *p* = 0.925, F = 0.009, ηp^2^ = 0.001 G × T: *p* = 0.483, F = 0.501, ηp^2^ = 0.012
Lumbar	-23.81 ± 8.14	-26.29 ± 8.25* d = 0.30	-26.00 ± 6.34	-27.10 ± 6.92 d = 0.17	T: *p* = 0.015, F = 6.462, ηp^2^ = 0.139 G × T: *p* = 0.332, F = 0.966, ηp^2^ = 0.024
Sacral	11.43 ± 5.62	13.86 ± 6.03* d = 0.41	12.86 ± 4.98	14.14 ± 5.17 d = 0.25	T: *p* = 0.013, F = 6.821, ηp^2^ = 0.146 G × T: *p* = 0.426, F = 0.646, ηp^2^ = 0.016
Inclination Angle	1.33 ± 2.45	1.52 ± 2.33 d = 0.08	0.33 ± 2.55	0.95 ± 2.85 d = 0.22	T: *p* = 0.216, F = 1.580, ηp^2^ = 0.038 G × T: *p* = 0.510, F = 0.443, ηp^2^ = 0.011
Sagittal Plane (Mobility)					
Thoracic	12.57 ± 14.23	23.00 ± 13.43* d = 0.75	18.19 ± 18.48	20.38 ± 17.17 d = 0.13	T: *p* = 0.003, F = 9.979, ηp^2^ = 0.200 G × T: *p* = 0.046, F = 4.253, ηp^2^ = 0.096
Lumbar	61.43 ± 18.60	69.86 ± 11.80* d = 0.49	65.29 ± 14.16	70.33 ± 13.82 d = 0.38	T: *p* = 0.015, F = 6.453, ηp^2^ = 0.139 G × T: *p* = 0.528, F = 0.406, ηp^2^ = 0.010
Sacral	66.57 ± 19.60	70.67 ± 14.10 d = 0.21	76.24 ± 10.01	53.48 ± 13.92* d = 1.80	T: *p* = 0.002, F = 10.425, ηp^2^ = 0.207 G × T: *p* < 0.001, F = 21.581, ηp2 = 0.350
Inclination Angle	123.00 ± 29.95	136.76 ± 11.07 d = 0.50	136.62 ± 13.01	118.43 ± 14.36* d = 1.31	T: *p* = 0.602, F = 0.277, ηp^2^ = 0.007 G × T: *p* < 0.001, F = 14.407, ηp^2^ = 0.265
Frontal Plane (Angle)					
Thoracic	7.00 ± 4.47	7.43 ± 3.62 d = 0.10	9.71 ± 3.08	7.90 ± 3.70* d = 0.53	T: *p* = 0.221, F = 1.545, ηp^2^ = 0.037 G × T: *p* = 0.051, F = 4.058, ηp^2^ = 0.092
Lumbar	7.81 ± 4.84	7.14 ± 3.15 d = 0.15	10.81 ± 3.80	8.48 ± 4.20* d = 0.63	T: *p* = 0.033, F = 4.875, ηp^2^ = 0.109 G × T: *p* = 0.227, F = 1.505, ηp^2^ = 0.036
Sacral	4.10 ± 2.80	3.67 ± 2.08 d = 0.14	5.67 ± 3.35	3.95 ± 2.03* d = 0.80	T: *p* = 0.031, F = 5.004, ηp^2^ = 0.111 G × T: *p* = 0.187, F = 1.801, ηp^2^ = 0.043
Inclination Angle	1.10 ± 1.22	1.10 ± 1.22 d = 0.00	1.24 ± 0.99	1.00 ± 0.89 d = 0.26	T: *p* = 0.601, F = 0.277, ηp^2^ = 0.007 G × T: *p* = 0.601, F = 0.277, ηp^2^ = 0.007
Frontal Plane (Mobility)					
Thoracic	79.48 ± 13.98	81.48 ± 15.53 d = 0.13	70.71 ± 15.77	68.38 ± 14.14 d = 0.15	T: *p* = 0.933, F = 0.007, ηp^2^ = 0.001 G × T: *p* = 0.279, F = 1.205, ηp^2^ = 0.029
Lumbar	48.38 ± 10.61	52.05 ± 12.10 d = 0.33	53.95 ± 10.58	46.48 ± 10.27* d = 0.74	T: *p* = 0.279, F = 1.206, ηp^2^ = 0.029 G × T: *p* = 0.003, F = 10.314, ηp^2^ = 0.205
Sacral	12.71 ± 4.42	11.00 ± 5.47 d = 0.32	10.38 ± 5.72	7.67 ± 4.24* d = 0.59	T: *p* = 0.014, F = 6.651, ηp^2^ = 0.143 G × T: *p* = 0.564, F = 0.339, ηp^2^ = 0.008
Inclination Angle	70.95 ± 8.57	72.76 ± 10.69 d = 0.20	69.24 ± 9.92	61.05 ± 11.43* d = 0.80	T: *p* = 0.093, F = 2.971, ηp^2^ = 0.069 G × T: *p* = 0.010, F = 7.296, ηp^2^ = 0.154
Matthias Test (Angle)					
Thoracic	-1.67 ± 4.76	-0.57 ± 5.15 d = 0.21	-0.90 ± 6.41	-1.76 ± 6.31 d = 0.14	T: *p* = 0.926, F = 0.009, ηp^2^ = 0.001 G × T: *p* = 0.448, F = 0.587, ηp^2^ = 0.014
Lumbar	-0.33 ± 4.31	-4.05 ± 4.28* d = 0.79	-0.62 ± 3.52	-2.95 ± 5.67 d = 0.43	T: *p* = 0.002, F = 10.543, ηp^2^ = 0.209 G × T: *p* = 0.463, F = 0.550, ηp^2^ = 0.014
Sacral	-0.48 ± 4.40	-3.38 ± 3.94* d = 0.70	-0.43 ± 4.10	-3.00 ± 6.10 d = 0.44	T: *p* = 0.014, F = 6.668, ηp^2^ = 0.143 G × T: *p* = 0.876, F = 0.025, ηp^2^ = 0.001
Inclination Angle	-1.10 ± 2.68	-7.38 ± 3.55* d = 1.97	-1.00 ± 1.76	-6.43 ± 4.64* d = 1.42	T: *p* < 0.001, F = 79.889, ηp^2^ = 0.666 G × T: *p* = 0.517, F = 0.428, ηp^2^ = 0.011
Thoracic Rotation Angle					
Right Thoracic Rotation Angle	74.14 ± 6.09	82.90 ± 6.18* d = 1.46	68.81 ± 7.00	69.90 ± 5.98 d = 0.15	T: *p* < 0.001, F = 64.815, ηp^2^ = 0.618 G × T: *p* < 0.001, F = 39.209, ηp^2^ = 0.495
Left Thoracic Rotation Angle	77.90 ± 7.90	85.86 ± 8.19* d = 1.00	74.57 ± 7.73	75.29 ± 7.21 d = 0.09	T: *p* < 0.001, F = 81.047, ηp^2^ = 0.670 G × T: *p* < 0.001, F = 56.530, ηp^2^ = 0.586

Two-way repeated-measures ANOVA results

*T* time effect, *G x T* group x time interaction effect, *F* *F*-value, *ηp*^*2*^ partial eta squared

*Significant difference from pre-test values (*p* < 0.05)

### Sagittal plane

#### Spinal angles

A significant main effect of time was observed for lumbar angle (T: *p* = 0.015, ηp² = 0.139) and sacral angle (T: *p* = 0.013, ηp² = 0.146). Post-hoc comparisons revealed significant increases in lumbar (-23.81 ± 8.14 to -26.29 ± 8.25) and sacral angles (11.43 ± 5.62 to 13.86 ± 6.03) in the exercise group only, whereas the control group showed no significant changes. Thoracic and inclination angles did not change significantly over time (*p* > 0.05). No significant group × time interactions were observed for any sagittal plane angles (*p* > 0.05).

#### Segmental mobility

Significant time effects were observed for thoracic (T: *p* = 0.003, ηp² = 0.200) and lumbar mobility (T: *p* = 0.015, ηp² = 0.139), with post-hoc analyses indicating significant increases only in the exercise group. Significant group × time interactions were found for sacral (G × T: *p* < 0.001, ηp² = 0.350), thoracic mobility (G × T: *p* = 0.046, ηp² = 0.096), and inclination mobility (G × T: *p* < 0.001, ηp² = 0.265), indicating significantly greater improvements in the exercise group compared to the control group.

### Frontal plane

#### Spinal angles

Significant time effects were found for lumbar (T: *p* = 0.033, ηp² = 0.109) and sacral angles (T: *p* = 0.031, ηp² = 0.111), with post-hoc analyses showing significant decreases in the exercise group only. Thoracic and inclination angles did not show significant changes over time. No significant group × time interactions were observed for any frontal plane angles.

#### Segmental mobility

Significant group × time interactions were found for lumbar (G × T: *p* = 0.003, ηp² = 0.205) and inclination mobility (G × T: *p* = 0.010, ηp² = 0.154), with the exercise group demonstrating greater improvements than the control group. Thoracic and sacral mobility did not show significant time or interaction effects.

### Matthias test

Significant time effects were observed for lumbar (T: *p* = 0.002, ηp² = 0.209), sacral (T: *p* = 0.014, ηp² = 0.143), and inclination angles (T: *p* < 0.001, ηp² = 0.666). Post-hoc analyses indicated significant decreases in these angles in the exercise group. No significant group × time interactions were detected.

### Thoracic rotation angles

Significant time effects were found for both right (T: *p* < 0.001, ηp² = 0.618) and left thoracic rotation angles (T: *p* < 0.001, ηp² = 0.670). Significant group × time interactions were also observed for right (G × T: *p* < 0.001, ηp² = 0.495) and left thoracic rotation angles (G × T: *p* < 0.001, ηp² = 0.586), indicating that the exercise group improved significantly more than the control group. Post-hoc tests confirmed that the exercise group showed substantial increases in both right and left thoracic rotation angles after six weeks.

### Summary of time and group × time effects

Overall, indicators showing a significant group × time interaction, and therefore more attributable to the thoracic exercise intervention, included thoracic, sacral, and inclination mobility in the sagittal plane, lumbar and inclination mobility in the frontal plane, and right and left thoracic rotation angles. In contrast, several outcomes showed a significant time effect only, including lumbar and sacral angles in both sagittal and frontal planes, lumbar mobility in the sagittal plane, sacral mobility in the frontal plane, and lumbar, sacral, and inclination angles during the Matthias test, suggesting changes over the training period that may reflect natural fluctuations rather than effects specifically attributable to the intervention (Table 3).

**Table 3:** Summary of variables showing group × time interaction versus time effect only

Effect type	Domain	Variables	Interpretation
Significant Group × Time (G × T) interaction	Segmental mobility - sagittal plane	Thoracic mobility ↑ Sacral mobility ↑ Inclination mobility ↑	Changes more attributable to the thoracic exercise intervention
	Segmental mobility - frontal plane	Lumbar mobility ↑ Inclination mobility ↑	Intervention-specific effects beyond routine training
	Thoracic rotation	Right thoracic rotation angle ↑ Left thoracic rotation angle ↑	Exercise-specific increases in rotational mobility
Significant Time effect only (no G × T interaction)	Spinal alignment - sagittal plane	Lumbar angle Sacral angle	Changes occurred over time in both groups
	Spinal alignment - frontal plane	Lumbar angle Sacral angle	Likely reflects natural fluctuations or routine training effects
	Segmental mobility	Lumbar mobility (sagittal plane) Sacral mobility (frontal plane)	Time-related changes without intervention specificity
	Matthias test	Lumbar angle ↓ Sacral angle ↓ Inclination angle ↓	Postural adaptations over the training period, not specifically attributable to the exercise program

### Effect sizes

Within-group effect sizes (Cohen’s d) ranged from small to very large. The largest effects were observed for thoracic rotation (d = 1.00–1.46) and Matthias test inclination angle (d = 1.42–1.97).

The sensitivity analysis using conservative baseline-value imputation yielded results consistent with the complete-case analysis. The primary outcome showed no significant group × time interaction, whereas significant group × time interactions for right and left thoracic rotation angles were preserved, with comparable effect sizes)(Supplementary Material 3).

## Discussion

This study examined the effects of adding thoracic spine exercises to routine training on spinal alignment, segmental mobility, and thoracic rotation in professional male soccer players. Thoracic spine exercises did not significantly alter sagittal thoracic curvature angle but led to meaningful improvements in spinal mobility and thoracic rotation, suggesting that such interventions may preferentially enhance dynamic spinal function rather than static alignment in professional soccer players. Specifically, thoracic spine exercises resulted in improvements in lumbar and sacral angles, as well as thoracic, lumbar, sacral, and inclination mobility in the sagittal plane, lumbar and sacral angles in the frontal plane, and bilateral thoracic rotation angles. Decreases (negative increases) were observed in lumbar, sacral, and inclination angles on the Matthias test. Compared with the control group, the exercise group demonstrated significantly greater increases in thoracic, sacral, and inclination mobility in the sagittal plane; lumbar and inclination mobility in the frontal plane; and bilateral thoracic rotation angles. Collectively, these findings suggest that thoracic spine exercises may support a holistic spinal adaptation that extends beyond local segments to include the lumbar and sacral regions.

In this study, thoracic spine exercises were found to be effective on functional parameters rather than on structural alignment. The fact that professional football players had baseline thoracic kyphosis values within physiological limits may be considered a possible factor explaining the absence of a marked change in thoracic alignment. Consistently, the literature reports that, in athletic populations with baseline kyphotic angles below 40°, exercises targeting the thoracic spine have limited capacity to modify structural curvatures, whereas more pronounced adaptations are observed in functional outcomes such as segmental mobility and rotational capacity [12, 35, 38, 58].

Studies on the effects of spinal exercise programs on spinal structure, segmental ROM, functional mobility, and performance have become a focus of recent research [14, 27, 28]. Exercises targeting the thoracic region, in particular, support segmental integrity of the spine and enhance in both the sagittal and frontal planes at a functional level [8, 12, 29]. Although the thoracic spine plays a key role in functional performance, few studies have specifically examined the effects of thoracic spine exercises on spinal alignment and mobility across different populations [12, 39]. For example, Leak examined the acute effects of thoracic spine exercises in healthy, physically active adults and reported improvements in thoracic mobility in both the sagittal and transverse planes [34]. Feng et al. demonstrated that a functional spinal exercise program applied twice weekly for eight weeks in patients with postural thoracic kyphosis increased thoracic ROM, thoracic curvature angle, and improved spinal inclination angle [35]. Similarly, Çelenay and Kaya observed decreases in thoracic and lumbar curvature angles in the sagittal plane following eight weeks of thoracic stabilization exercises [12]. Choi et al. also reported acute increases in thoracic ROM in healthy adults receiving thoracic spine exercises [37]. Yang et al. found that thoracic mobilization and stabilization exercises performed over 12 weeks increased thoracic ROM in the sagittal plane [36]. Le Solliec et al. reported that exercise training for the spine and shoulder girdle, including thoracic spine exercises, did not improve the thoracic curvature angle in the sagittal plane, but increased thoracic mobility in tennis players [38]. Patel and Parulekar observed improvements in thoracic mobility in swimmers following thoracic spine exercises [39]. Overall, these studies indicate that thoracic spine exercises can improve thoracic flexion–extension ROM in both athletes and other populations. The increases in thoracic mobility in the sagittal plane observed in our study are therefore consistent with the current literature.

The physiological curvatures of the spine can change over time due to various factors [59]. Athletic performance often involves trunk flexion and extension in the sagittal and frontal planes, as well as rotational movements around the longitudinal axis [60]. Repetitive, demanding, and intensive training loads athletes are exposed to over years of training play an important role in the adaptation of spinal morphology [60, 61]. Accordingly, several studies have investigated the effects of certain postural positions on spinal curvatures in sports requiring high spinal mobility, such as volleyball, wrestling, skiing, soccer, and rowing [62–65]. In particular, a relationship has been reported between the development of thoracic kyphosis and upright postures in sports involving frequent forward bending [64]. Physiologically, the thoracic kyphosis angle is generally considered to range between 20° and 40°, while values exceeding 40°–45° are classified as increased thoracic kyphosis [12, 66]. Studies have shown that exercises performed in the sagittal plane can reduce the thoracic curvature angle, particularly in non-athlete individuals with thoracic kyphosis angles of 44° or higher [12, 35]. Conversely, interventions applied to athletes with thoracic kyphosis angles below 40° have not been found to produce significant changes in the curvature angle [38]. In our study, the mean baseline thoracic kyphosis angle of the soccer players who received thoracic spine exercises was 39.71°, and the exercise protocol did not result in a significant change in the thoracic curvature angle in either the sagittal or frontal planes. However, a significant between-group difference emerged in the frontal plane, which appears to be related to postural changes in the control group rather than a direct effect of the intervention. These findings suggest that the effect of thoracic spine exercises on thoracic curvature may depend on baseline postural characteristics and initial kyphosis angle.

Thoracic mobility has been shown to support athletic performance by enhancing force production, movement transfer, and biomechanical efficiency, particularly in sports involving repetitive upper extremity movements [39, 67]. In this context, exercises targeting the thoracic region may contribute to improvements in athletic performance. However, since sports performance and injury incidence were not directly assessed in the present study, it is not possible to associate the observed findings with improvements in performance or reductions in injury risk. Nevertheless, the increases in segmental mobility and thoracic rotation observed may represent potential biomechanical mechanisms capable of supporting functional movement quality in sports such as football, which require high rotational capacity and multiplanar movement patterns. Further studies incorporating direct performance metrics and injury-related outcomes are needed to clarify this relationship.

Thoracic spine mobility in football plays an important role in the effective control of trunk kinematics during high-intensity movements such as sprinting, rapid changes of direction, jumping, passing, and shooting [17, 18]. In these complex movements, the transfer of force generated by the lower extremities to the trunk and upper segments via ground reaction forces depends on the thoracic spine having sufficient range of motion in the sagittal and frontal planes [8, 19–22]. The increases in thoracic mobility observed in the present study may be considered a functional adaptation that supports the force transfer mechanisms and trunk control required for sport-specific movements in football players. This finding further corroborates the structural importance of thoracic spine mobility within the kinematic chain for the dynamic kinematics of football. The increases in lumbar and sacral mobility observed in our study highlight the indirect benefits of thoracic exercises on lower spinal segments. Thoracic spine mobility is essential for coordinated movement of the spinal segments, and restrictions in this region may lead to compensatory movements in other parts of the spine or kinetic chain [67–69]. Therefore, thoracic spine exercises may also promote functional improvements in the lower spinal segments. Feng et al. reported that corrective functional exercises improved spinal inclination angle, but no differences were observed compared with the control group in either inclination angle or mobility [35]. In our study, thoracic spine exercises resulted in increased inclination mobility in both sagittal and frontal planes. These findings suggest that thoracic spine exercises can enhance trunk control not only at a segmental level but also across multiple planes, supporting coordinated movement of the spine and contributing to more efficient, integrated motor control. However, due to the limited number of studies on this topic, further research is needed to clarify these effects.

The Matthias test, designed to evaluate spinal postural stabilization and adaptive musculoskeletal responses under load, is widely used as a functional assessment of posture. However, studies applying the Matthias test in athletes are relatively limited. Feng et al. reported that after an 8-week functional spinal exercise program, no significant changes were observed in the thoracic and lumbar angles during the Matthias test, but a negative increase was found in the sacral and trunk inclination angles [35]. Similarly, Zawadka et al. observed a decrease in thoracic angle, an increase in lumbar lordosis, and a negative increase in trunk inclination angle during the Matthias test in young adults [58]. In our study, the exercise group showed no significant changes in thoracic angle values on the Matthias test, although a tendency towards mild kyphosis was noted. Additionally, decreases were observed in the lumbar, sacral and trunk inclination angles, reflecting adaptive postural responses. However, no significant differences were found when compared with the control group. Previous research has indicated that positioning the arms at 90° flexion increases thoracic stabilization, and a small increase in thoracic kyphosis in healthy individuals during this position is considered a physiological adaptation [70]. It has been reported that forward arm elevation shifts the center of gravity anteriorly, resulting a partial increase in lumbar lordosis to maintain postural balance. This adaptive mechanism helps maintain stability during loading, provided the lordotic increase remains within physiological limits. Excessive increases, however, may increase the risk of developing hyperlordosis [70, 71]. The anterior increase in trunk inclination observed in our study is consistent with these literature findings, supporting the concept of physiological adaptation during forward arm elevation.

The thoracic spine plays a critical role in maintaining optimal biomechanical alignment in sports due to its contribution to trunk rotation [7, 8]. In sports that involve extensive trunk rotation, the mobility of the thoracic segments is a key factor in enhancing performance and reducing injury risk [30]. Soccer, in particular, requires substantial thoracic spine mobility because of the high levels of trunk rotation and frequent sudden changes of direction inherent to the sport [72]. However, the findings of the present study cannot be directly interpreted as evidence of performance enhancement or a reduction in injury risk, and this relationship requires confirmation in future studies. Optimal rotation of the thoracic spine and trunk segments facilitates force transfer during actions such as shooting and passing, increasing ball speed and passing intensity and contributing to overall performance [17, 73]. Studies evaluating thoracic spine rotation angles indicate that thoracic spine exercises increase rotational ROM [32, 34, 37, 39, 41]. Consistent with these findings, the results of our study suggest that thoracic spine exercises can provide functional benefits by increasing thoracic rotation in sports where trunk rotation is essential, such as soccer. From an anatomical and biomechanical perspective, thoracic facet joints are oriented in the coronal plane to provide stability against anterior translation, while the lower thoracic segments limit rotation depending on lumbar orientation [74, 75]. The rib cage, including the sternum, ribs, joints, ligaments, and intervertebral discs, further constrains thoracic ROM, particularly axial rotation [10, 11, 76, 77]. In many sports, the thoracic spine and surrounding joint segments contribute to the movements of the shoulder complex [78, 79]. During movements requiring high rotation, such as shooting, adequate thoracic rotation and extension improve movement efficiency and reduce stress on the glenohumeral joint [80, 81]. Conversely, thoracic rotation limitations can lead to compensatory movement strategies, increasing injury risk [78]. Therefore, improvements in thoracic rotation play a critical role in enhancing functional performance and preventing injuries related to rotational stress.

To better understand which changes can be attributed specifically to the intervention, both effects over time and group × time interactions were examined. The time effect (T) indicates whether a variable changed from pre- to post-test across all participants, regardless of group assignment. The group × time interaction (G × T) reflects whether the change over time differed between the exercise and control groups, which is the primary indicator of intervention effectiveness. In our study, thoracic rotation angles showed significant G × T interactions, indicating that increases in rotational mobility were specific to the exercise group and not observed in controls, supporting the functional benefits of thoracic spine exercises. In contrast, variables such as lumbar and sacral sagittal angles showed significant time effects without corresponding G × T interactions, suggesting these changes occurred over time but were not uniquely attributable to the exercise intervention. These results highlight that the observed improvements in thoracic rotation are likely a direct effect of the intervention, whereas changes in other spinal segments may reflect natural adaptation or variability over time. To facilitate interpretation, Table 3 summarizes variables showing intervention specific group x time effects versus those demonstrating time effects only.

The contribution of this study to the literature lies in being one of the few randomized controlled trials to investigate the effects of thoracic spine exercises on spinal alignment and segmental mobility in professional soccer players. The multidimensional assessment of the spine across the sagittal, frontal, and transverse planes enabled a more comprehensive evaluation of the multiplanar effects of thoracic-focused exercises. Moreover, the practical feasibility of the applied thoracic spine exercise protocol under field conditions provides important applied value, as it allows the findings to be directly integrated into training processes. In this respect, the present study offers an original and practically applicable contribution to the literature regarding the functional effects of thoracic spine exercises in professional soccer players. Furthermore, the findings of this study provide a basis for future research on thoracic spine interventions in athletic populations.

Because attrition was low and balanced between groups, and sensitivity analyses under conservative missing-data assumptions did not materially alter the results, the risk of attrition-related bias is considered minimal. Compared with the complete case analysis, conservative imputation led to small reductions in F statistics and effect sizes for thoracic rotation outcomes, while all significant effects were preserved.

The present study has some limitations. First, the inclusion of only healthy, professional male soccer players limits the generalizability of the findings to athletes of different genders, age groups, competitive levels, or sports disciplines. While the assessment tools used in the study were field-based, practical, and applicable, offering a methodological advantage, the absence of imaging-based methods (e.g., magnetic resonance imaging or posture analysis systems) limited the ability to evaluate structural changes in the spine in detail. The thoracic spine exercise protocol employed in this study was developed based on existing literature and clinical practices; however, the limited number of standardized protocols for thoracic spine exercises may have constrained the design of the intervention. Finally, the potential effects of spinal changes on sports performance and injury prevention were not examined, and is considered a limitation of the study. Further studies incorporating direct performance measures, injury-related outcomes, and longer follow-up periods are needed to clarify the sport-specific relevance of these functional adaptations.

## Conclusion

The addition of a six-week thoracic spine exercise program to routine soccer training did not result in significant changes in sagittal thoracic kyphosis in professional male soccer players. However, the intervention produced significant improvements in functional spinal parameters, particularly thoracic rotation and selected measures of segmental mobility, beyond those observed with routine training alone. These findings suggest that thoracic spine–focused exercise programs may preferentially enhance dynamic spinal function rather than static alignment in athletes. The applied intervention was feasible, well tolerated, and safely integrated into the competitive training environment. Based on these findings, coaches, sports science specialists, and physiotherapists may consider incorporating thoracic spine exercises into training and/or rehabilitation programs to support spinal function, enhance trunk rotation, and potentially contribute to improved athletic performance.

## Supplementary Information

Supplementary Material 1.

Supplementary Material 2.

Supplementary Material 3.

## Data Availability

The individual de-identified participant data supporting the findings of this study, along with the data dictionary and statistical analysis code, are available from the corresponding author upon reasonable request.

## Abbreviation

ROM: Range of Motion
