# Supplementary material for: Effects of adding thoracic spine exercises to routine soccer training on spinal alignment and mobility in professional male soccer players: a randomized controlled study
Source: BMC Sports Sci Med Rehabil. 2026 Mar 12;18:207. doi: 10.1186/s13102-026-01633-9 (PMC13094134; doi:10.1186/s13102-026-01633-9)
Supplement: Supplementary file 1 — Supplementary Material 1. [file 13102_2026_1633_MOESM1_ESM.docx]

# **Appendix A. Thoracic Spine Exercise Training Manual**

## General Instructions

All exercises should be performed in a **controlled, pain-free manner**. Movements should be smooth and deliberate, avoiding jerky or forced motions. Participants should breathe normally throughout the exercises and avoid holding their breath. Exercise intensity should remain at a **moderate level** (Borg RPE 11–13). Rest **30–45 seconds between sets** and **30–60 seconds between exercises**.

## **1. Thoracic Extension with a Foam Roller**

Begin by lying on your back with your knees bent and feet flat on the floor. Place the foam roller horizontally under your upper back (thoracic spine), just below the shoulder blades. Support your head and neck by gently interlocking your fingers behind your head, without pulling on the neck.

Slowly lean your upper back backward over the foam roller, allowing your chest to open while keeping your lower back and hips controlled. Pause briefly at the end range, then return to the starting position.

Perform the movement in a slow and controlled manner, focusing on extension through the thoracic spine rather than the lumbar region.

**Repetitions and progression:**

- Weeks 1–2: 2 sets of 10 repetitions
- Weeks 3–4: 3 sets of 10 repetitions
- Weeks 5–6: 3 sets of 15 repetitions

## **2. Thoracic Extension Flexibility Exercise**

Begin in a kneeling position with both knees on the floor. Place your elbows on a bench or elevated surface positioned in front of you, keeping your forearms supported.

From this position, gently lower your chest toward the floor by moving your trunk forward, allowing the thoracic spine to extend. Hold the end position briefly while maintaining a comfortable stretch, then return to the starting position.

Ensure that the movement remains controlled and pain-free throughout the exercise.

**Repetitions and progression:**

- Weeks 1–2: 2 sets of 10 repetitions
- Weeks 3–4: 3 sets of 10 repetitions
- Weeks 5–6: 3 sets of 15 repetitions

## **3. Thoracic Spine Rotation Exercise (Plank Position)**

Begin in a plank position with your body aligned and your weight supported evenly. Keep one arm firmly on the ground to stabilize your trunk.

With the free arm, reach underneath your body toward the opposite side, allowing gentle trunk rotation. Then lift the same arm upward and backward, rotating your thoracic spine while keeping your pelvis and lower body stable. Follow the moving hand with your head and eyes.

Return to the starting position and repeat the movement.

**Repetitions and progression:**

- Weeks 1–2: 2 sets of 8 repetitions
- Weeks 3–4: 3 sets of 10 repetitions
- Weeks 5–6: 3 sets of 12 repetitions

## **4. Thoracic Rotation in Quadrupedal Position**

Begin on all fours with your hands under your shoulders and knees under your hips. Place one hand behind your neck while keeping the other hand on the floor for support.

From this position, rotate your upper body by bringing your elbow downward toward the supporting arm. Then rotate upward by lifting the elbow toward the ceiling, focusing on movement through the thoracic spine.

Keep your pelvis stable and avoid shifting your weight excessively.

**Repetitions and progression:**

- Weeks 1–2: 2 sets of 8 repetitions
- Weeks 3–4: 3 sets of 10 repetitions
- Weeks 5–6: 3 sets of 12 repetitions

## **5. Side-Plank Thoracic Rotation (Open Book)**

Begin by lying on your side and assume a side-plank position supported on the lower arm, ensuring that the shoulder, trunk, and lower extremities are aligned. Extend the upper arm straight upward toward the ceiling. While maintaining stability of the lower body and pelvis, slowly move the upper arm forward across the front of the torso and underneath the chest. Then, rotate the arm upward and backward, opening the chest toward the ceiling through controlled thoracic rotation. Allow the head and eyes to follow the moving hand throughout the movement. Pause briefly at the end range, then return the arm to the starting position in a controlled manner.

From Week 3 onward, elastic resistance bands are added to increase difficulty as motor control improves.

**Repetitions and progression:**

- Weeks 1–2: 2 sets of 8 repetitions
- Weeks 3–4: 2 sets of 10 repetitions with a light elastic resistance band
- Weeks 5–6: 2 sets of 12 repetitions with a moderate elastic resistance band

## **6. Bird-Dog Exercise**

Begin on all fours with your spine in a neutral position. Engage your trunk muscles to maintain stability.

Slowly extend one arm forward while simultaneously extending the opposite leg backward. Hold briefly while maintaining balance and trunk control, then return to the starting position. Alternate sides with each repetition.

From Week 3 onward, elastic resistance bands are introduced to increase challenge.

**Repetitions and progression:**

- Weeks 1–2: 2 sets of 8 repetitions
- Weeks 3–4: 2 sets of 10 repetitions with a light elastic resistance band
- Weeks 5–6: 2 sets of 12 repetitions with a moderate elastic resistance band
