# Supplementary material for: Effects of adding thoracic spine exercises to routine soccer training on spinal alignment and mobility in professional male soccer players: a randomized controlled study
Source: BMC Sports Sci Med Rehabil. 2026 Mar 12;18:207. doi: 10.1186/s13102-026-01633-9 (PMC13094134; doi:10.1186/s13102-026-01633-9)
Supplement: Supplementary file 2 — Supplementary Material 2. [file 13102_2026_1633_MOESM2_ESM.docx]

**Table S1.** Thoracic Spine Exercise Training Program (TIDieR-compliant intervention description)

| **Exercise** | **Sets × repetitions (baseline)** | **Progression across 6 weeks** | **Intensity (RPE)** | **Rest intervals** |
| --- | --- | --- | --- | --- |
| Thoracic spine extension exercise with a foam roller | 2 × 10 | Weeks 1–2: 2 × 10 Weeks 3–4: 3 × 10 Weeks 5–6: 3 × 15 | 11–13 | 30–45 s between sets |
| Thoracic extension flexibility exercise | 2 × 10 | Weeks 1–2: 2 × 10 Weeks 3–4: 3 × 10 Weeks 5–6: 3 × 15 | 11–13 | 30–45 s between sets |
| Thoracic spine rotation exercise | 2 × 8 | Weeks 1–2: 2 × 8 Weeks 3–4: 3 × 10 Weeks 5–6: 3 × 12 | 11–13 | 30–45 s between sets |
| Thoracic rotation exercise in quadrupedal position | 2 × 8 | Weeks 1–2: 2 × 8 Weeks 3–4: 3 × 10 Weeks 5–6: 3 × 12 | 11–13 | 30–45 s between sets |
| Open book exercise | 2 × 8 | Weeks 1–2: 2 × 8 Weeks 3–4: 2 × 10 + light elastic band Weeks 5–6: 2 × 12 + moderate elastic band | 11–13 | 30–45 s between sets |
| Bird-dog exercise | 2 × 8 | Weeks 1–2: 2 × 8 Weeks 3–4: 2 × 10 + light elastic band Weeks 5–6: 2 × 12 + moderate elastic band | 11–13 | 30–45 s between sets |

**Notes:**

- Exercises were performed three times per week for six weeks (total of 18 sessions).
- 30–60 s rest was allowed between exercises.
- Exercise progression was based on individual adaptation, maintenance of correct movement form, and perceived exertion.
- Exercise intensity was monitored using the Borg Rating of Perceived Exertion (RPE) scale.
- All participants completed the intervention with 100% adherence; missed sessions were rescheduled within the same week.
