# Supplementary material for: Effects of adding thoracic spine exercises to routine soccer training on spinal alignment and mobility in professional male soccer players: a randomized controlled study
Source: BMC Sports Sci Med Rehabil. 2026 Mar 12;18:207. doi: 10.1186/s13102-026-01633-9 (PMC13094134; doi:10.1186/s13102-026-01633-9)
Supplement: Supplementary file 3 — Supplementary Material 3. [file 13102_2026_1633_MOESM3_ESM.docx]

****Table S2.**** Sensitivity analysis of spinal alignment and thoracic rotation outcomes using baseline observation carried forward imputation

|  | **Exercise Group (n=22)** | | **Control Group (n=22)** | |  |
| --- | --- | --- | --- | --- | --- |
|  | **Pre-test**  **Mean ± SD** | **Post-test**  **Mean ± SD** | **Pre-test**  **Mean ± SD** | **Post-test**  **Mean ± SD** | **Two-way ANOVA (T and G x T):**  **p, F, ηp^2^** |
| **Sagittal Plane (Angle)** |  |  |  |  |  |
| Thoracic | 39.77 ± 6.35 | 40.36 ± 8.60  d = 0.07 | 42.45 ± 7.93 | 41.68 ± 7.53  d = 0.10 | T: p = 0.925, F = 0.009, ηp^2^ = 0.001  G × T: p = 0.483, F = 0.502, ηp^2^ = 0.012 |
| **Thoracic Rotation Angle** |  |  |  |  |  |
| Right Thoracic Rotation Angle | 74.32 ± 6.00 | 82.68 ± 6.12*  d=1.38 | 68.86 ± 6.84 | 69.91 ± 5.83  d = 0.16 | T: p < 0.001, F = 58.095, ηp^2^ = 0.580  G × T: p < 0.001, F = 35.144, ηp^2^ = 0.456 |
| Left Thoracic Rotation Angle | 78.09 ± 7.76 | 85.68 ± 8.04*  d=0.96 | 74.64 ± 7.55 | 75.32 ± 7.04  d = 0.09 | T: p < 0.001, F = 70.249, ηp^2^ = 0.626  G × T: p < 0.001, F = 48.999, ηp^2^ = 0.538 |

Two-way repeated-measures ANOVA results. T: time effect, G x T: group x time interaction effect; F: F-value, ηp^2^: partial eta squared. *Significant difference from pre-test values (p < 0.05).
